# The association of host and vector characteristics with *Ctenocephalides felis* pathogen and endosymbiont infection

**DOI:** 10.3389/fmicb.2023.1137059

**Published:** 2023-03-06

**Authors:** Charlotte Moore, Edward B. Breitschwerdt, Lisa Kim, Yiyao Li, Kelli Ferris, Ricardo Maggi, Erin Lashnits

**Affiliations:** ^1^Intracellular Pathogens Research Laboratory, Comparative Medicine Institute, College of Veterinary Medicine, North Carolina State University, Raleigh, NC, United States; ^2^Department of Medical Sciences, School of Veterinary Medicine, University of Wisconsin, Madison, WI, United States; ^3^Department of Clinical Sciences, College of Veterinary Medicine, North Carolina State University, Raleigh, NC, United States

**Keywords:** *Bartonella*, flea, *Wolbachia*, vector phylogenetics, flea associated *Rickettsia*, host-vector agreement, cat flea

## Abstract

Surveillance of the fleas and flea-borne pathogens infecting cats is important for both human and animal health. Multiple zoonotic *Bartonella* and *Rickettsia* species are known to infect the most common flea infesting cats and dogs worldwide: *Ctenocephalides felis*, the cat flea. The ability of other flea species to transmit pathogens is relatively unexplored. We aimed to determine cat host and flea factors independently associated with flea *Bartonella* and *Rickettsia* infection. We also assessed flea and cat infection by flea-host pair and location. To accomplish these aims, we performed qPCR for the detection of *Bartonella*, hemotropic *Mycoplasma*, *Rickettsia*, and *Wolbachia* DNA using paired cat and flea samples obtained from free-roaming cats presenting for spay or neuter across four locations in the United States. A logistic regression model was employed to identify the effect of cat (sex, body weight, geographic location, and *Bartonella*, hemotropic *Mycoplasma*, and *Rickettsia* spp., infection) and flea (clade and *Rickettsia* and *Wolbachia* infection) factors on *C*. *felis Bartonella clarridgeiae* infection. From 189 free roaming cats, we collected 84 fleas: *Ctenocephalides felis* (78/84), *Cediopsylla simplex* (4/84), *Orchopeas howardi* (1/84), and *Nosopsyllus fasciatus* (1/84). *Ctenocephalides felis* were phylogenetically assigned to Clades 1, 4, and 6 by *cox1* gene amplification. *Rickettsia asembonensis* (52/84) and *B*. *clarridgeiae* (16/84) were the most common pathogenic bacteria detected in fleas. Our model identified host cat sex and weight as independently associated with *B*. *clarridgeiae* infection in fleas. *Rickettsia asembonensis* (52/84), *Rickettsia felis* (7/84) and *Bartonella henselae* (7/84) were detected in specific clades: *R*. *felis* was detected only in Clades 1 and 6 while *B*. *henselae* and *R*. *asembonensis* were detected only in Clade 4. *Wolbachia* spp., also displayed clade specificity with strains other than *Wolbachia* wCfeT only infecting fleas from Clade 6. There was poor flea and host agreement for *Bartonella* spp., infection; however, there was agreement in the *Bartonella* species detected in cats and fleas by geographic location. These findings reinforce the importance of considering reservoir host attributes and vector phylogenetic diversity in epidemiological studies of flea-borne pathogens. Widespread sampling is necessary to identify the factors driving flea-borne pathogen presence and transmission.

## 1. Introduction

*Ctenocephalides felis*, the cat flea, is the most common ectoparasite of the domestic dog and cat worldwide (Rust, 2017). Other flea species such as *Cediopsylla simplex*, *Ctenocephalides canis*, *Echidnophaga gallinacea*, *Nosopsyllus fasciatus*, and *Pulex* spp., are occasionally reported as ectoparasites of the domestic cat (Akucewich et al., 2002; Thomas et al., 2016; Abdullah et al., 2019). Little is known about the disease-causing capacity of these other flea species as a primary cause of skin irritation or a vector for pathogen transmission. The cat flea alone has the capacity to vector multiple zoonotic organisms (*Bartonella* spp., *Dipylidium caninum*, *Rickettsia* spp.; Rust, 2017). The factors influencing cat flea *Bartonella* and *Rickettsia* infection are currently unknown but have important implications for risk assessment and disease epidemiology in animals and humans.

The three *Bartonella* spp., associated with *C*. *felis* and their cat hosts are *Bartonella henselae*, *Bartonella clarridgeiae*, and *Bartonella koehlerae* (Chomel et al., 1996; Kordick et al., 1997; Avidor et al., 2004; Taber et al., 2022). Infection with these species is a cause of both animal and human disease with numerous manifestations including endocarditis, nervous system, rheumatological, and vascular diseases (Breitschwerdt, 2017; Álvarez-Fernández et al., 2018; Canneti et al., 2019). Studies analyzing the *Bartonella* spp., infection status of cats and their fleas generally report poor agreement between cat and flea pairs (Chomel et al., 1996; La Scola et al., 2002; Gutiérrez et al., 2015; Mifsud et al., 2020; Azrizal-Wahid et al., 2021). The role of host factors (e.g., body weight, sex) in association with flea *Bartonella* spp., infection were not investigated in these studies. One study investigating *C*. *felis* phylogenetic diversity and pathogen infection failed to identify associations with specific haplotypes (Azrizal-Wahid et al., 2021). Global sampling and *cox*1 gene sequencing by Lawrence et al. identified 8 distinct, bioclimatically limited *C*. *felis* clades (Lawrence et al., 2019). These include the temperate (Clades 1 and 2), tropical (Clades 3–6), and African (Clades 7 and 8) clades. A limited number of studies have utilized these established clades to shed light on flea *Bartonella* spp., infection: *B*. *clarridgeiae* infection has been reported in Clades 1, 3, 4, and 6, *B*. *henselae* infection has been reported in Clades 1, 4, and 6, and *B*. *koehlerae* infection has been reported in Clades 1 and 4 (Šlapeta and Šlapeta, 2016; Chandra et al., 2017; Manvell et al., 2022).

*Rickettsia felis*, *Rickettsia asembonensis*, and *Candidatus* ‘Rickettsia senegalensis’ comprise a group known as the *Rickettsia felis*-like organisms (RFLO) which are known to be vectored by *C*. *felis* (Reif and Macaluso, 2009; Legendre and Macaluso, 2017; Maina et al., 2019). Clinical signs of RFLO infection in humans are generally non-specific, and similar to those caused by other *Rickettsia* spp., such as acute headache, nausea, pyrexia, rash, and muscle, back, and joint pain (Richter et al., 2002; Richards et al., 2010). RFLO are occasionally detected in cats (Mullins et al., 2018; Phoosangwalthong et al., 2018; Maina et al., 2019) however previous studies suggested that the domestic dog or the flea could serve as a reservoir host (Barrs et al., 2010; Hii et al., 2011; Ng-Nguyen et al., 2020). Phylogenetic studies have identified *R*. *asembonensis* infection in Clade 4, and *R*. *felis* infection in Clades 1 and 6 (Manvell et al., 2022). Clade 3 *C*. *felis* were infected with an unconfirmed RFLO (Šlapeta and Šlapeta, 2016). Otherwise, there is limited information on the risk factors for flea infection with RFLO.

Hemotropic *Mycoplasma* species (hMyc) are occasionally, but not always detected in the cat flea and the cat flea has been proposed as a potential vector for hMyc (Lappin et al., 2006; Barrs et al., 2010; Persichetti et al., 2016; Abdullah et al., 2019). However, in laboratory experiments *C*. *felis* did not transmit hMyc efficiently (Woods et al., 2006) and other studies have suggested fighting as a mechanism of transmission among cats and potentially to humans (Barker, 2019; Hattori et al., 2020; Tasker, 2022). The manifestation of hMyc infection in animals and humans appears to primarily be pyrexia and hemolytic anemia with the ability to cause severe and potentially life-threatening disease in a subset of cases (Barker, 2019; Hattori et al., 2020; Tasker, 2022).

Another flea-associated genus of interest is *Wolbachia*, which infect a majority of arthropod and helminth species and manipulate insect reproduction, vector competence, and vector efficiency on a strain specific basis (Werren et al., 2008). Three *Wolbachia* strains have been isolated from *C*. *felis:* wCfeF, wCfeJ, and wCfeT (Driscoll et al., 2020; Khoo et al., 2020). Limited research has investigated the *C*. *felis* associated *Wolbachia* strains and their effect on vector and pathogen success, despite representing a potential opportunity for vector and/or pathogen control, as has been accomplished in mosquitos (Dorigatti et al., 2018). Investigating coinfection of *Wolbachia* and pathogenic *Bartonella* or *Rickettsia* spp., in *C*. *felis* may be important for flea-borne pathogen epidemiology. Microbiome analysis has revealed widespread coinfection of *C*. *felis* with *Bartonella*, *Rickettsia*, and *Wolbachia*, but this observation does not eliminate the possibility that a specific *Wolbachia* strain may impact vector pathogen acquisition, maintenance, and/or transmission in *C*. *felis* (Manvell et al., 2022).

Given the existing knowledge gaps surrounding flea-borne pathogen coinfection and associations with flea and reservoir host factors, we aimed to identify the cat (sex, body weight, geographic location, and *Bartonella*, hemotropic *Mycoplasma*, and *Rickettsia* spp., infection) and flea (phylogenetic clade and *Bartonella*, *Rickettsia*, and *Wolbachia* co-infection) factors influencing *Bartonella* and *Rickettsia* presence in fleas collected from free-roaming cats. We tested three specific hypotheses regarding the most common flea *Bartonella* spp., *B*. *clarridgeiae*: (1) location and flea clade are independently associated with flea *B*. *clarridgeiae* infection, (2) fleas collected from *B*. *clarridgeiae* infected cat hosts are more likely to be infected with *B*. *clarridgeiae*, and (3) flea coinfection with *Rickettsia* and *Wolbachia* spp., is associated with flea *B*. *clarridgeiae* infection.

## 2. Materials and methods

### 2.1. Sample collection

With the assistance of veterinarians and staff at partner Trap-Neuter-Release (TNR) programs, fleas and cat tissue were collected at four locations in North Carolina, Virginia, and Wisconsin under North Carolina State University IACUC protocol #19–003 and #21–468, and University of Wisconsin IACUC protocol #V006461. Samples collected from Washington, NC were obtained through collaboration with Paws and Love, Inc. in March 2019. Collections from Raleigh, NC were performed at the North Carolina State University Veterinary Hospital in April through June 2022. Collections in Orange, VA were performed at Paradocs Animal Hospital with the assistance of the Orange County Humane Society TNR program in May 2019. Collections from Madison, WI were performed in collaboration with the Madison Cat Project in March through August 2021. Cats presenting for routine spay and neuter through each local TNR program were selected for participation regardless of sex or apparent flea presence. Cats were excluded if eartip tissue was not obtained.

In order to collect fleas, volunteers were instructed to comb all cats regardless of apparent parasites or flea dirt with special attention paid to the tail base and ventral regions. Each cat was assigned their own flea comb and collection bag into which combings were placed. Fleas and combs were then frozen until the flea species was identified and DNA extracted.

Sample size calculations were based on pilot data on the proportion of cats with fleas able to be collected (estimated 40%) and estimating that approximately 50% of fleas would have PCR amplifiable *Bartonella* spp., DNA or *Rickettsia* spp., DNA. Logistically, using our sampling design with partner TNR organizations, it appeared feasible to collect samples from approximately 40 cats over a 1–6 month period. Therefore, with a total sample of 160 cats and 80 fleas (40 cats and 20 fleas per sampling location), we would have 80% power to detect an odds ratio of 4 or greater between the proportion of fleas with and without pathogen DNA and proposed binary explanatory variables (cat sex, cat infection with *Bartonella* spp., flea pathogen co-infection, flea *Wolbachia* co-infection). This was expected to be a large enough difference to be clinically and practically relevant for this exploratory study.

### 2.2. Fleas

Upon receiving fleas at the NCSU Intracellular Pathogens Research Laboratory, fleas were identified to the species level with the assistance of Dr. James Flowers, Clinical Professor of Parasitology (North Carolina State University, College of Veterinary Medicine). Fleas were individually washed and crushed according to previously reported protocol (Manvell et al., 2022). DNA was then extracted utilizing the Qiagen DNeasy Blood & Tissue Kit (Qiagen, Valencia, CA, United States) following the manufacturer’s tissue extraction protocol. DNA concentration and purity were determined spectrophotometrically (Thermo Fisher Scientific, Waltham, MA, United States). DNA from 18% (15/84) of fleas used for this study’s sample was also used for a previously published manuscript analyzing the *C*. *felis* microbiome (Manvell et al., 2022).

### 2.3. Tissues

When presenting for spay and neuter, free-roaming cats regularly have one eartip removed while under anesthesia to allow them to be visually identified as spayed/neutered following release. Instead of discarding the eartip, we collected and froze the tissue which was later dissected utilizing a scalpel and forceps disinfected with 94% ethanol between each sample. DNA extraction from tissues was performed utilizing the Qiagen DNeasy Blood & Tissue Kit (Qiagen, Valencia, CA, United States) following the manufacturer’s protocol. DNA concentration and purity were determined spectrophotometrically (Thermo Fisher Scientific, Waltham, MA, United States). DNA from eartip tissue from 39% (73/189) of the cats reported in this study, including 32% (17/53) of flea infested cats, were previously published in a manuscript comparing the presence of flea-borne pathogens in eartip and reproductive tissues (Manvell et al., 2021).

### 2.4. Polymerase chain reaction

Quantitative real-time PCR (qPCR) for *Bartonella*, *Rickettsia*, hMyc, and *Wolbachia* spp., was performed utilizing the primers listed below (Table 1). Following genus level qPCR, positive samples underwent Sanger sequencing. For these sequences, species and strain identity was determined by alignment with NCBI Basic Local Alignment Search Tool (BLAST). If 23S-5S *Rickettsia* qPCR returned a sequence that was not readable (e.g., overlapping peaks, inappropriate length) or had an inappropriate melting temperature, *R*. *felis* and *R*. *asembonensis* specific primers targeting the *ompA* gene were employed to confirm the infecting species with an expected length of 222 and 183 base pairs, respectively. Primer development was performed in AlignX utilizing *R*. *asembonensis* (GenBank MK923742.1) and *R*. *felis* (GenBank MG818714.1). Specificity was determined by *in silico* analysis with comparison to 22 *Rickettsia* spp., including *Rickettsia rickettsii* (DQ002504.1), *Rickettsia parkeri* (U43801.1), and *Rickettsia rhipicephali* (U43803.1). Primers were validated with *C*. *felis* samples of known infection status including eight *R*. *asembonensis* and eight *R*. *felis* infected *C*. *felis*, and numerous uninfected *C*. *felis*.

**Table 1:** Primers targeting housekeeping genes and pathogens including primer name, sequence, gene target, and reference to conditions.

**Target organism**	**Oligonucleotide name**	**Oligonucleotide sequence (5′-3′)**	**Target gene**	**Reference**
*Bartonella* spp.	Bart_ssrA_F	GCTATGGTAATAAATGGACAATGAAATAA	*ssrA*	Tyrrell et al. (2019)
	Bart_ssrA_R3	GACGTGCTTCCGCATAGTTGTC		
*Bartonella* spp.	BsppITS325s	CCTCAGATGATGATCCCAAGCCTTCTGGCG	ITS	Maggi et al. (2020)
	BsppITS543as	AATTGGTGGGCCTGGGAGGACTTG		
	BsppITS500p	FAM-GTTAGAGCGCGCGCTTGATAAG-IABkFQ		
*Rickettsia* spp.	Rick23-5_F2	AGCTCGATTGATTTACTTTGCTG	23S-5S	Tyrrell et al. (2019)
	Rick23-5_R	CCACCAAGCTAGCAATACAAA		
*Rickettsia felis*	RifelisOmpA-172 s	AGTCCTTGGTGCTGCAAGAACCGTAACTG	*ompA*	This study
	RifelisOmpA-330as	ACCACTGAACCTAATGAAATATCACCAGT		
*Rickettsia asembonensis*	RiasemboOmpA-175 s	GTTGGGAGGAACAACGATAGATGCA	*ompA*	This study
	RiasemboOmpA-245as	ACCGTAAATAAACCAGGAGCAAAACCA		
*Mycoplasma* spp.	Myco_Hf_F.1	GACGAAAGTCTGATGGAGCAAT	16S rRNA	Manvell et al. (2021)
	Myco_Hf_R	ACGCCCAATAAATCCGRATAAT		
*Wolbachia* spp.	AE16S_45F	AGCYTAACACATGCAAGTCGAACG	16S rRNA	Tyrrell et al. (2020)
	AE16S_299R	CCTCTCAGACCAGCTATAGATCA		
*Ctenocephalides felis*	Cff-F	AGAATTAGGTCAACCAGGA	*cox1*	Lawrence et al. (2014)
	Cff-R	GAAGGGTCAAAGAATGATGT		
Flea species	LCO1490	GGTCAACAAATCATAAAGATATTGG	*cox1*	Folmer et al. (1994)
	HCO2198	TAAACTTCAGGGTGACCAAAAAATCA		

GADPH, glyceraldehyde-3-phosphate dehydrogenase, ITS, intergenic spacer, FAM, 6-fluorescein amidite; IABkFQ, IowaBlack®FQ.

Flea phylogeny was assessed by conventional PCR amplification of the *cox1* gene. *Ctenocephalides felis* DNA was amplified using the Cff-F and Cff-R primers (Lawrence et al., 2014) while other flea species were amplified using the LCO1490 and HCO2198 primers (Folmer et al., 1994).

Each run included two negative controls: a No Template Control (NTC) consisting of nuclease free water and DNA from cat blood confirmed to be negative for the genus of interest. Each run also included a positive control plasmid. The *Bartonella* spp., intergenic spacer (ITS) qPCR utilized *B*. *henselae* culture as a positive control. Negative control DNA and positive control plasmids were obtained from the Vector Borne Disease Diagnostics Lab (VBDDL) at North Carolina State University.

### 2.5. Statistical methods

Data handling was performed in R version 4.1.0 (R Foundation for Statistical Computing, Vienna, Austria) using the here (Müller, 2020), janitor (Firke, 2021), reshape2, stats, stringr, and tidyverse (Wickham et al., 2019; Wickham, 2021) packages. Phylogenetic analysis was performed in R using the ape (Paradis and Schliep, 2019), bios2mds, Biostrings (Pagès et al., 2021), haplotypes, ips, irr (Gamer et al., 2019), msa (Bodenhofer et al., 2015), pegas (Paradis, 2010; Tsirogiannis and Sandel, 2016, 2017), PhyloMeasures, and seqinr packages. Visualization was performed in R using the cowplot (Wilke, 2020), ggsci (Xiao, 2018), ggplot2 (Wickham, 2016), and ggpubr packages. Fisher’s exact tests were employed to assess five different associations between categorical variables. Comparisons included: (1) Proportion of fleas with any *Bartonella* spp., by geographic location (4 locations); (2) proportion of fleas with any *Rickettsia* spp., by geographic location (4 locations); (3) proportion of fleas with any *Wolbachia* spp., by geographic location (4 locations); (4) proportion of fleas with *R*. *asembonensis* by presence of any *Bartonella* spp (any *Bartonella* spp., present/absent); (5) proportion of fleas with any *Bartonella* spp., by presence of any *Wolbachia* spp (any *Wolbachia* spp., present/absent). *p* values <0.05 were considered significant; due to the exploratory nature of this analysis and to prevent overly conservative *p* values, correction for multiple comparisons was not performed.

A multivariable logistic regression model was developed to identify the variables associated with *B*. *clarridgeiae* infection in *Ctenocephalides felis* fleas. Variables considered for inclusion are shown in Table 2, and included geographic location, cat hMyc, cat *Bartonella*, flea clade, and flea *Rickettsia* infection as categorical variables, cat sex and flea *Wolbachia* infection as binary variables, and cat weight as a continuous variable. Variables were first compared individually with the outcome of interest (flea *B*. *clarridgeiae* infection) *via* univariate logistic regression model and those with a *p*-value less than 0.25 were selected for inclusion in the preliminary model. The number of fleas collected from each flea’s host was included to control for the collection of multiple fleas from the same host. Additional models were created which systematically removed and replaced individual variables. These additional models were compared to the preliminary model on the basis of Akaike information criterion (AIC) and a Hosmer-Lemeshow Goodness of Fit test (GOF). When the removal of a variable resulted in a significantly different fit (GOF *p* < 0.05) or reduced AIC that variable was retained in the final model. The odds ratio (OR) and 95% confidence interval is reported for variables selected for the final model.

**Table 2:** Summary of sampling based on geographic location of origin including the dates of sampling, total number of cats, number of flea infested cats, total number of fleas, median number of fleas per infested cat (and range), and summary of non-*Ctenocephalides felis* sampled fleas.

**Location**	**Date**	**Cats**	**Flea infested cats (%)**	**Fleas collected**	**Median fleas per cat (range)**	**Non-***Ctenocephalides felis* **fleas (number)**
Raleigh, NC	04–2022 to 06–2022	57	25 (44%)	36	1 (1–5)	N/A
Washington, NC	03–2019	63	11 (17%)	15	1 (1–3)	N/A
Orange, VA	12–2020 to 01–2021	41	8 (20%)	15	1.5 (1–4)	N/A
Madison, WI	04–2021 to 08–2021	28	9 (32%)	18	1 (1–10)	*Cediopsylla simplex* (4) *Nosopsyllus fasciatus* (1) *Orchopeas howardi* (1)
**Total**		**189**	**53 (28%)**	**84**	**1 (1–10)**	**N/A**

These flea species include *Cediopsylla simplex*, *Nosopsyllus fasciatus*, and *Orchopeas howardi*. N/A indicates that category is not applicable to a given location. Bolded values represent the totals across locations.

Following selection of the final model, additional models were created to test three specific hypotheses: (1) location and flea clade are independently associated with flea *B*. *clarridgeiae* infection, (2) fleas collected from *B*. *clarridgeiae* infected cat hosts are more likely to be infected with *B*. *clarridgeiae*, and (3) flea coinfection with *Rickettsia* and *Wolbachia* spp., is associated with flea *B*. *clarridgeiae* infection. These models included the selected variables for the final model, the number of fleas per host, and the variables of interest in each hypothesis.

## 3. Results

In total we obtained a tissue sample from 189 cats, 53 of which had one or more fleas collected. The number of fleas and cats sampled at each location is shown in Table 2.

### 3.1. Flea characteristics and PCR results

**Figure 1:**
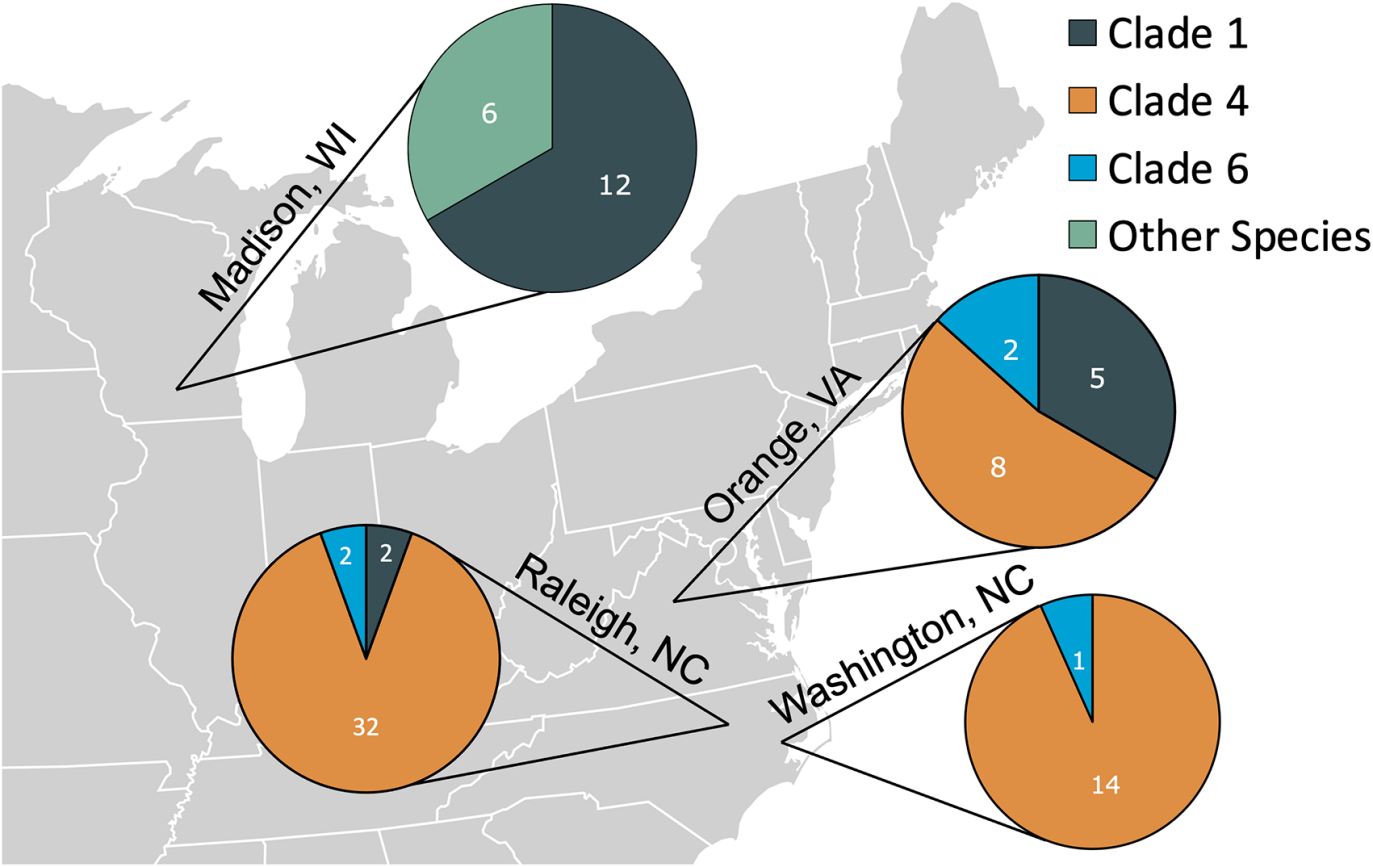
The number of fleas from each location assigned to the *Ctenocephalides felis* clades defined by Lawrence et al. (2019) or belonging to other flea species *(Cediopsylla simplex*, *Orchopeas howardi*, and *Nosopsylus fasciatus)*.

By far the most common flea species collected was *C*. *felis* (93%, 78/84); however, other flea species were collected from cats in Madison, WI including *Cediopsylla simplex* (5%, 4/84), *Orchopeas howardi* (1/84), and *Nosopsyllus fasciatus* (1/84). No cat was infested with more than one flea species. The *C*. *felis* collected during this study were assigned to Clades 1, 4, and 6, as defined by Lawrence et al. (2019). The proportion of each *C*. *felis* clade by location are displayed in Figure 1. *Cediopsylla simplex* fleas were assigned to three haplotypes (Accession ID: OP713785, OP713787, OP713885) with no homologous submissions in GenBank (421/427, 98.5%, Accession ID: HM398833.1). The one *N*. *fasciatus cox*1 sequence (Accession ID: OP713901) was novel with only 86% (358/417) homology with *N*. *fasciatus* sequences from Belgium (Accession ID: LT158040). The one *O*. *howardi* flea (Accession ID: OP737457) displayed 99% (423/427) homology to *Orchopeas caedens* (Accession ID: HM398830.1) in the absence of available *O*. *howardi* sequences for comparison.

**Figure 2:**
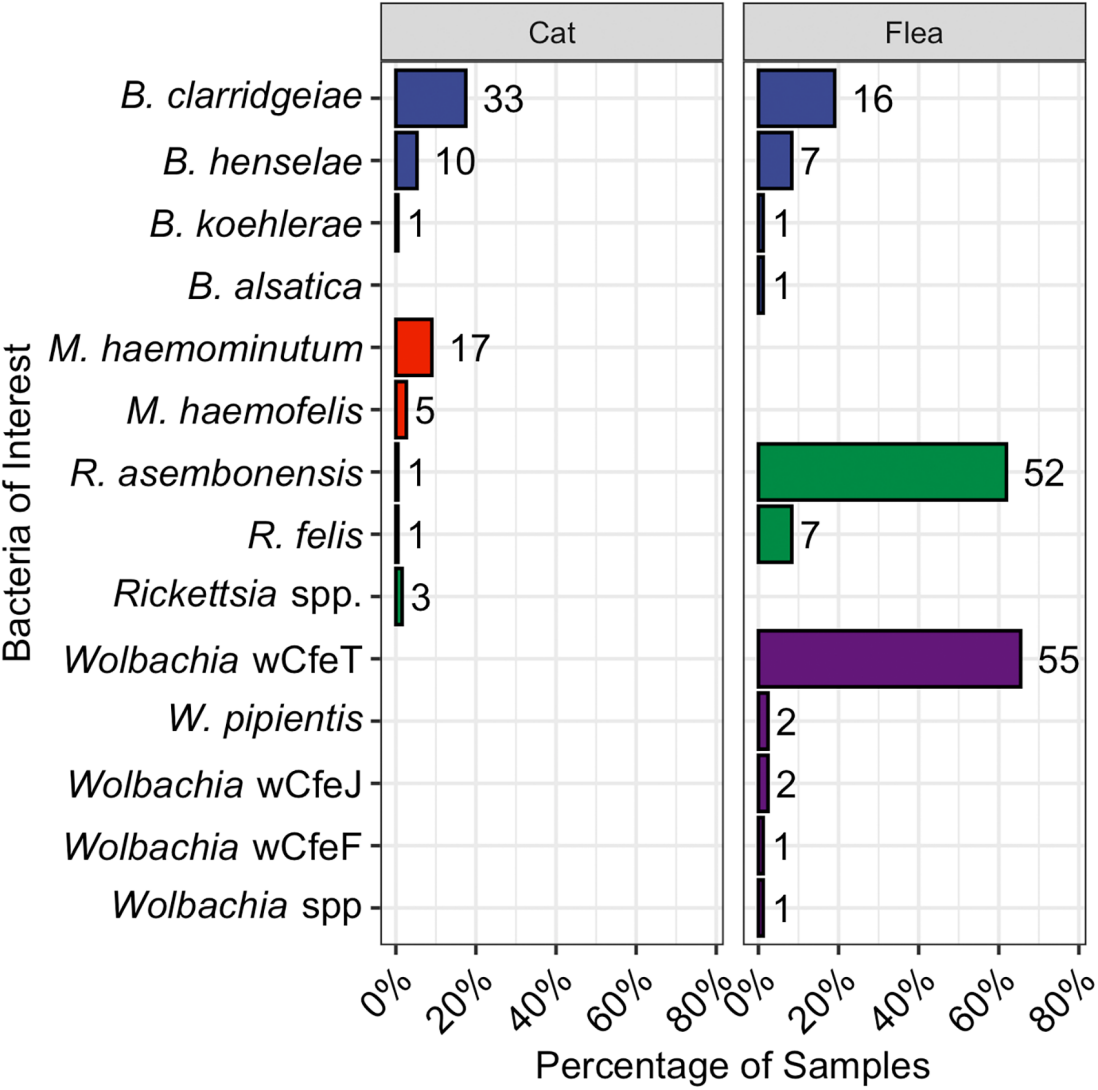
Bar chart displaying the proportion of cats (left) or fleas (right) infected with the various pathogens and bacteria as reported by qPCR. The total number is displayed to the right of each bar. *Bartonella* spp., are in blue, hemotropic *Mycoplasma* spp., in red, *Rickettsia* spp., in green, and *Wolbachia* spp., in purple.

The percentage of fleas infected with each bacterial genus is shown in Figure 2. A single *C*. *felis* contained a *Wolbachia* spp., that was distinct from previously described strains (Accession ID: OP731570). The *O*. *howardi* (*n* = 2) and *N*. *fasciatus* (*n* = 1) fleas were infected with *Wolbachia pipientis*, or a strain not able to be differentiated over the amplified region of the 16S gene. Hemotropic *Mycoplasma* spp., DNA was not amplified from fleas of any species.

### 3.2. Cat characteristics and PCR results

Female (51%, 97/189) and male (49%, 92/189) cats were sampled approximately equally. The percentage of cats infected with each bacterial genus is shown in Figure 2. *Bartonella* spp., were the most common flea-borne pathogen detected in the cats. Five cats (all from Raleigh, NC) were infected with *Rickettsia* spp. This includes two previously undescribed *Rickettsia* spp. amplicons with 100% homology over the 283 base pair sequence (Accession ID: OP744991). The closest available GenBank match was *R*. *felis* (Accession ID: KJ796446.1) with low query cover. Attempts to amplify a product using *R*. *felis* ompA specific primers were unsuccessful. The second unknown *Rickettsia* spp., was amplified from a single cat (Accession ID: OP744990) and most closely aligned with *Rickettsia* spp. isolates from *Ixodes scapularis* in New York (221/226, 98%, Accession ID: MN704870.1) and *Amblyomma americanum* in New York and North Carolina (221/226, 98%, Accession ID: KJ796407.1; Lee et al., 2014).

### 3.3. Flea-cat PCR agreement

**Figure 3:**
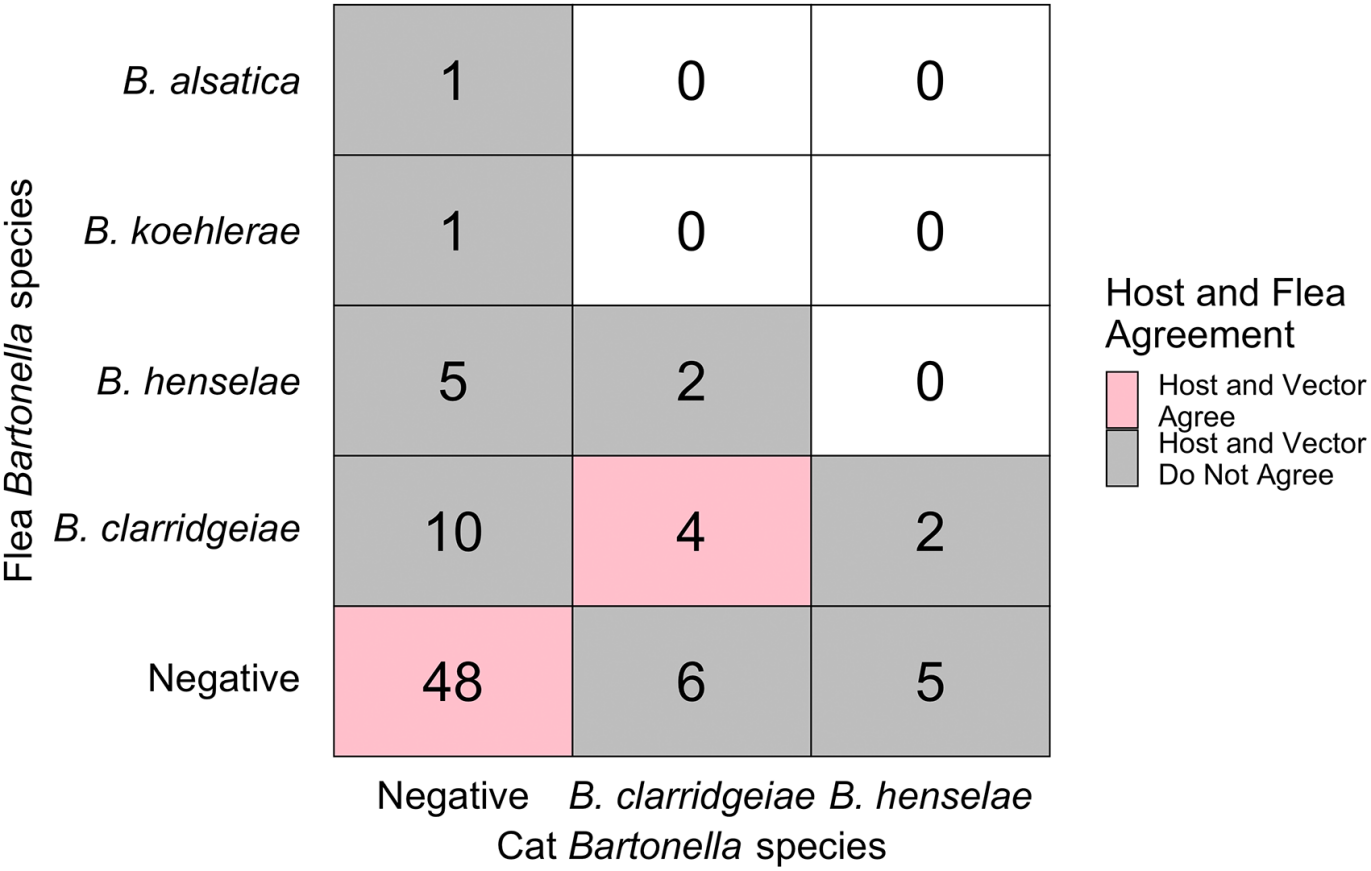
Chart displaying the number of fleas infected with each *Bartonella* spp. (*y*-axis) and their host’s infection status (*x*-axis). Pink indicates host-vector agreement while grey indicated host-vector disagreement regarding pathogen infection status. Number indicates the number of fleas (not cats) in each category.

**Figure 4:**
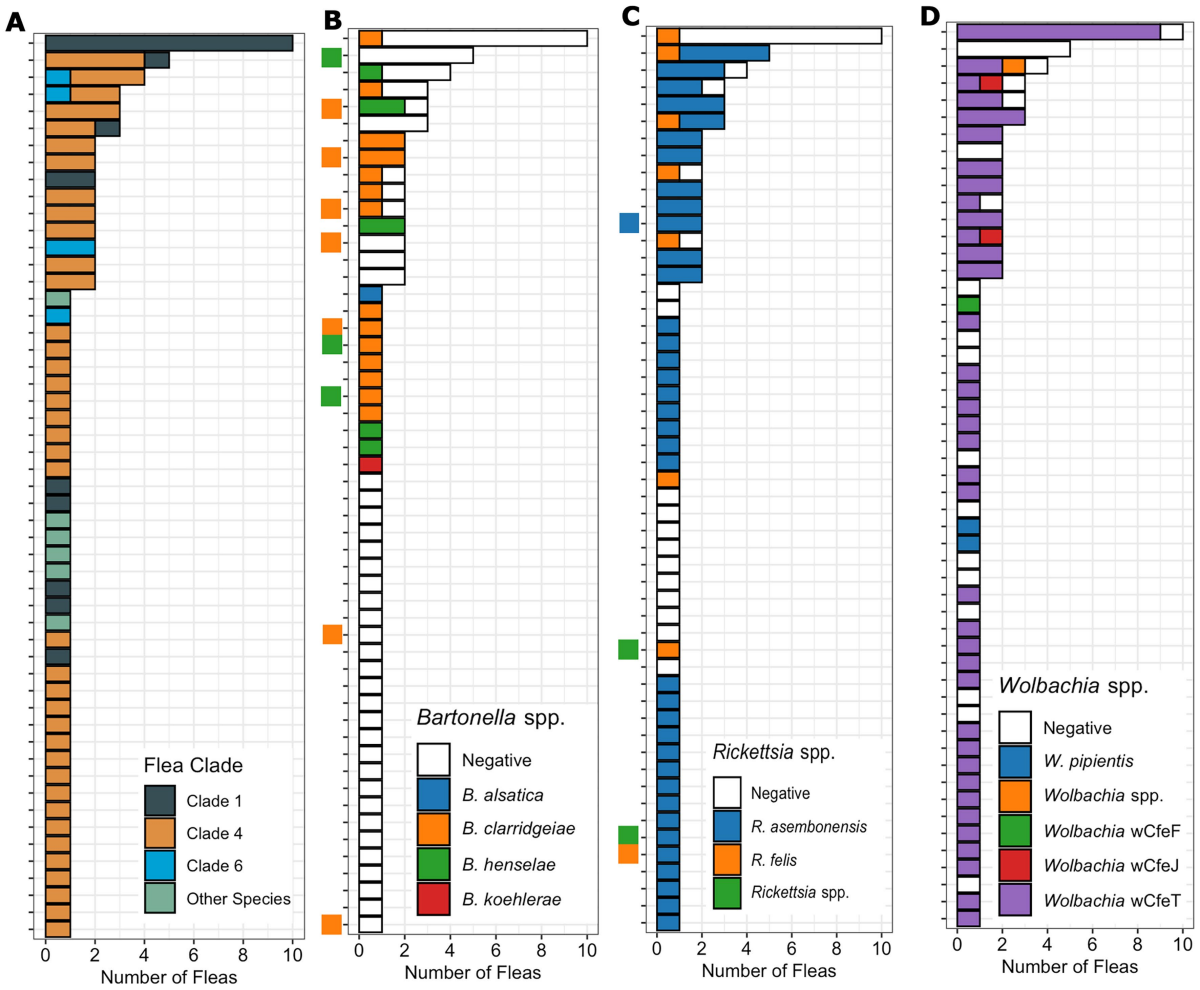
Bar chart displaying the number of fleas (x-axis) from each host (y-axis) by flea species or clade **(A)** as well as the *Bartonella* **(B)**, *Rickettsia* **(C)**, and *Wolbachia* **(D)** species or strains identified by qPCR. The cat host *Bartonella* **(C)** and *Rickettsia* **(D)** are indicated by a small square on the y-axis with uninfected cats left blank. The cat host is kept consistent across figure sections to allow comparison on a specific cat host.

Nineteen percent (10/53) of flea infested cats were infected with *Bartonella* spp. Of these 10 *Bartonella* spp. infected cats with fleas, 6 yielded one or more flea that was infected with a *Bartonella* spp.; however, that flea often had a different *Bartonella* spp. based upon PCR amplification and sequencing than the host cat (3/6, Figures 3, 4B). A majority of *Bartonella* spp. infected fleas were collected from *Bartonella* spp., qPCR negative cats (68%, 17/25, Figure 3).

Nine percent (5/53) of flea infested cats were infected with *Rickettsia* spp. A majority of *Rickettsia* spp. infected fleas were collected from *Rickettsia* spp. qPCR negative cats (68%, 54/80, Figure 4C). All five *Rickettsia* spp. infected cats yielded one or more *Rickettsia* spp. infected flea, but only one of these cats had the same *Rickettsia* spp. in tissue and flea based on PCR amplification and sequencing.

Hemotropic *Mycoplasma* DNA was amplified from cats, but not from fleas. *Wolbachia* spp. DNA was amplified only from fleas, but not from cats.

There were 15 cats with more than one flea collected; flea clade, *Bartonella* spp. *Rickettsia* spp. and *Wolbachia* spp. PCR results from each flea is shown in Figure 4. For cats with multiple fleas, infestation by *C*. *felis* fleas from multiple clades was common (27%, 4/15, Figure 4A). Infestation of a cat with fleas containing different *Bartonella* spp. was not detected (Figure 4B). In cats with more than one flea collected, often only a single flea was infected with *Bartonella* spp. (6/10). In contrast, for *Rickettsia* and *Wolbachia* spp., cats with multiple fleas frequently had multiple fleas positive (Figures 4C,D). Twelve cats (80%) had multiple fleas infected with *Rickettsia* spp., and twelve cats (80%) had multiple fleas infected with *Wolbachia* spp. Infestation with fleas infected with different *Rickettsia* spp. (2 cats) or *Wolbachia* (3 cats) strains on the same cat was also detected.

### 3.4. Associations between pathogens in fleas and explanatory factors

**Figure 5:**
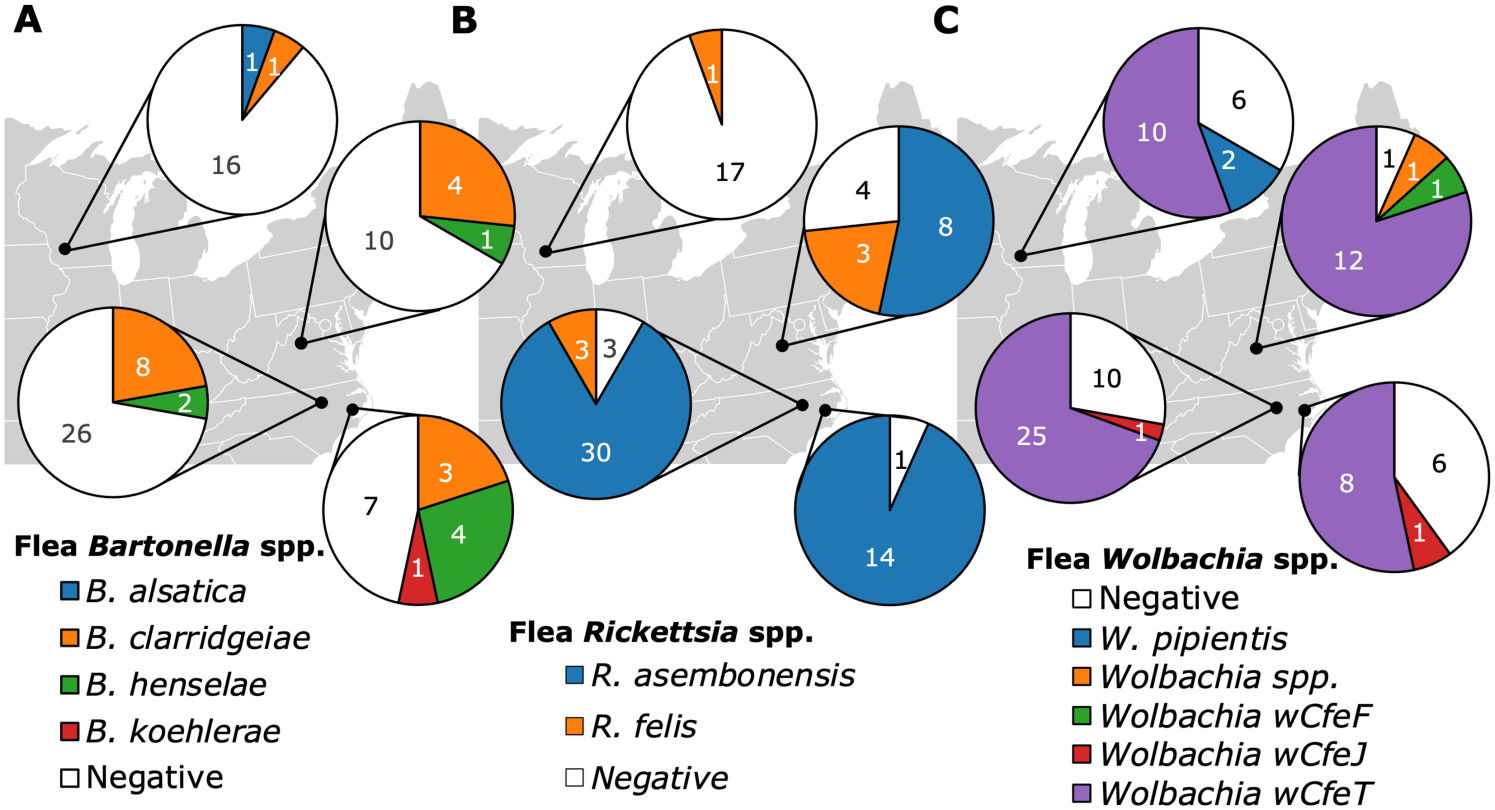
Pie charts displaying the *Bartonella* **(A)**, *Rickettsia* **(B)**, and *Wolbachia* **(C)** infection status of fleas based on their geographic location of origin including Raleigh NC, Washington NC, Orange VA, and Madison WI.

Infection with *Bartonella* in fleas, considering all three species together in the analysis, was not significantly associated with geographic location (*p* = 0.068, Figure 5A). When aggregated by geographic location, the same *Bartonella* spp. were found in cats and fleas, except for *B*. *alsatica* that was only found in one *C*. *simplex* (Supplementary Figure S1) and no cats in Madison WI. Furthermore, the relative proportion of *Bartonella* spp. agreed between sample types (flea and cat) at all locations except for Washington, NC, where *B*. *henselae* prevalence exceeded *B*. *clarridgeiae* in fleas, but not in cats (Supplementary Figure S1). In contrast, *Rickettsia* spp. infection in fleas, considering all species together, was significantly associated with geographic location (*p* < 0.0001, Figure 5B). *Wolbachia* infection in fleas was not associated with geographic location (*p* = 0.43, Figure 5C).

**Figure 6:**
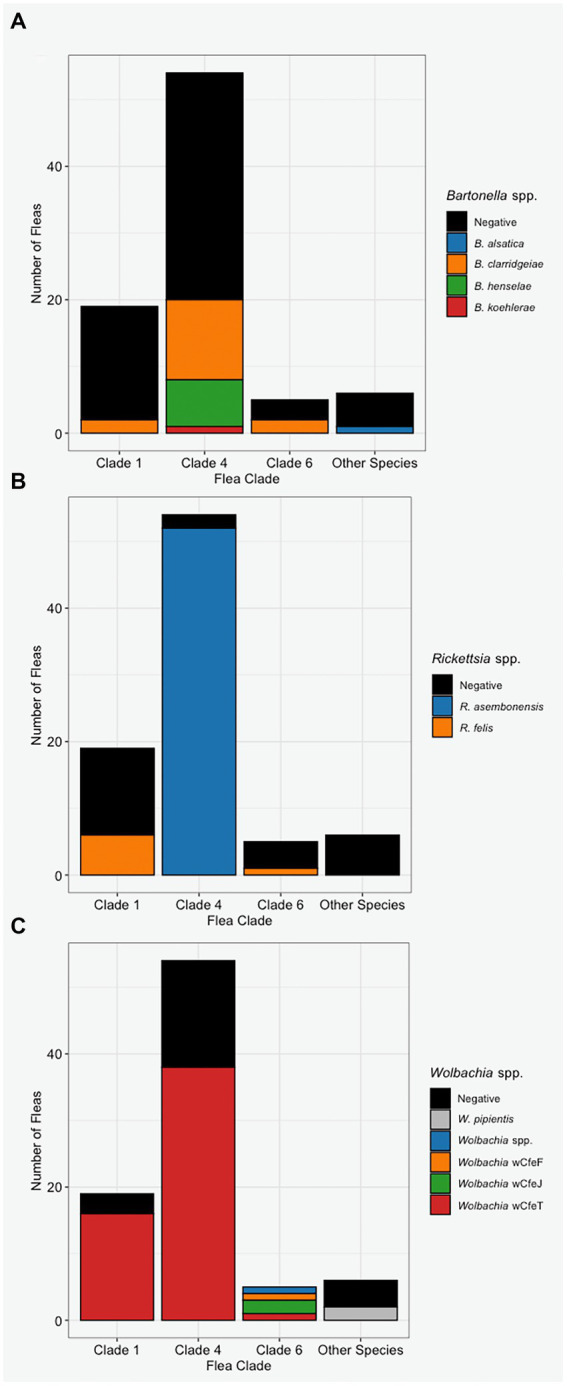
The *Bartonella* **(A)**, *Rickettsia* **(B)**, and *Wolbachia* **(C)** infection status of fleas based on their assigned clade or species (*x*-axis).

*Bartonella clarridgeiae* was the only *Bartonella* spp. detected in more than one *C*. *felis* clade as *B*. *henselae* and *B*. *koehlerae* were only detected in Clade 4 (Figure 6A), however infection with the genus *Bartonella* was not associated with *C*. *felis* clade (*p* = 0.28). *Bartonella alsatica* was detected only in a single *C*. *simplex*. *Rickettsia* spp. were strictly clade specific. *Rickettsia asembonensis* was detected in almost all fleas from Clade 4 (96%, 52/54) and *R*. *felis* was detected only in Clades 1 (32%, 6/19) and 6 (20%, 1/5, Figure 6B; *p* < 0.0001). *Wolbachia* spp. also displayed clade specificity with *Wolbachia* strain wCfeT being the only strain found in *C*. *felis* Clades 1 and 4 (Figure 6C). Despite being represented by the smallest sample size, Clade 6 *C*. *felis* had the greatest diversity of *Wolbachia* strains including strains wCfeF, wCfeJ, wCfeT, and a previously undescribed *Wolbachia* strain. The association of *Rickettsia* and *Wolbachia* with *C*. *felis* clade persisted even when fleas from diverse clades were infesting the same cat (Figure 4).

**Figure 7:**
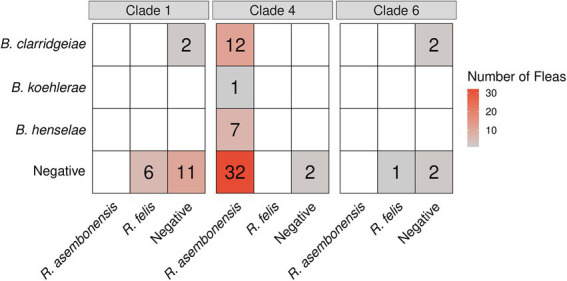
Heatmap displaying the *Bartonella* (*y*-axis) and *Rickettsia* (*x*-axis) coinfection status by *Ctenocephalides felis* clade.

*Bartonella* spp. in fleas was associated with *R*. *asembonensis* coinfection (*p* = 0.030). Coinfected fleas included all *B*. *henselae* (*n* = 7) and *B*. *koehlerae* (*n* = 1), and a majority of *B*. *clarridgeiae* infected *C*. *felis* (75%, 12/16, Figure 7). No *R*. *felis* infected flea (*n* = 7) was coinfected with a *Bartonella* spp. A majority of *Bartonella* spp. infected fleas were also coinfected with *Wolbachia* spp. (68%, 17/25); however, this was not statistically significant compared to the proportion of all fleas infected with *Wolbachia* spp. (71%, 60/84; *p* = 0.79).

A logistic regression model was developed to investigate potential independent associations between explanatory factors and flea *B*. *clarridgeiae* infection. Univariate associations with each variable of interest were calculated (Table 3), and the final multivariable model showed that cat sex and body weight were independently associated with *C*. *felis B*. *clarridgeiae* infection (Table 4). A higher proportion of male cats had fleas with *B*. *clarridgeiae* than female cats (OR 4.22, 95% CI 1.13–15.7), and for each 1 kg increase in cat host body weight, fleas had 0.41 lower odds of *B*. *clarridgeiae* infection (95% CI 0.17–0.98). When controlling for cat body weight, sex, and the number of fleas collected per cat, geographic location and flea clade were not associated with *B*. *clarridgeiae* infection of *C*. *felis* (Table 5). Similarly, when controlling for cat body weight, sex, and the number of fleas collected per cat, neither cat *B*. *clarridgeiae* infection nor flea infection status was associated with *B*. *clarridgeiae* infection of *C*. *felis* (Table 4).

**Table 3:** Summary of the variables considered for inclusion in the flea *B*. *clarridgeiae* model.

**Considered variables**		**All fleas**	*Bartonella clarridgeiae*	**Infected fleas**
		**Total (%)**	**Total (%)**	***P*-value**
Host sex*				
	Male Intact	26 (33%)	9 (56%)	0.03
	Female Intact	52 (67%)	7 (44%)	Ref
Host weight (kg)*				
Median (Range)		3.20 (1.20–5.70)	2.79 (1.2–4.50)	0.09
Host *Bartonella* spp.*				
	*Bartonella clarridgeiae*	12 (15%)	4 (25%)	0.20
	*Bartonella henselae*	7 (9%)	2 (12%)	0.46
	Negative	59 (76%)	10 (62%)	Ref
**Host hemotropic *Mycoplasma* spp.**				
	*Mycoplasma haemominutum*	5 (6%)	1 (6%)	0.95
	*Mycoplasma haemofelis*	2 (3%)	0 (0%)	0.99
	Negative	71 (91%)	15 (94%)	Ref
**Geographic location**				
	Raleigh, NC	36 (46%)	8 (50%)	Ref
	Washington, NC	15 (19%)	3 (19%)	0.86
	Orange, VA	15 (19%)	4 (25%)	0.73
	Madison, WI	12 (15%)	1 (6%)	0.31
Number of fleas*				
Median (Range)		2 (1–10)	2 (1–10)	0.19
**Flea *Bartonella* spp.**				
	*Bartonella clarridgeiae*	16 (21%)		
	*Bartonella henselae*	7 (9%)		
	*Bartonella koehlerae*	1 (1%)		
	Negative	54 (69%)		
**Flea *Rickettsia* spp.**				
	*Rickettsia asembonensis*	52 (67%)	12 (75%)	0.86
	*Rickettsia felis*	7 (9%)	0 (0%)	0.99
	Negative	19 (24%)	4 (25%)	Ref
**Flea *Wolbachia* spp.**				
	*Wolbachia* spp.	58 (74%)	11 (69%)	0.57
	Negative	20 (26%)	5 (31%)	Ref
**Flea clade**				
	Clade 1	19 (24%)	2 (12%)	0.28
	Clade 4	54 (69%)	12 (75%)	Ref
	Clade 6	5 (6%)	2 (12%)	0.38

Variables were tested *via* univariate logistic modeling. All variables other than weight were reported as the total number and percentage of *C*. *felis* fleas in each category. Weight was reported as the median and range of cat host weight. Shaded areas indicate that the variable is synonymous with the variable we were modeling and thus not considered. Selection for inclusion in the preliminary model is indicated by *.

**Table 4:** Summary of the model selected to predict *B*. *clarridgeiae* infection in *Ctenocephalides felis* including only the variables significantly associated with host and flea characteristics and potentially confounding variable (number of fleas per host cat).

		**Estimate**	***P-*value**	**OR (95% CI)**
***Bartonella clarridgeiae*** **flea infection model**				
Cat sex	Female	Ref	Ref	Ref
	Male	1.44	0.032*	4.22 (1.13–15.70)
Cat weight		−0.89	0.046*	0.41 (0.17–0.98)
Number of fleas		−0.08	0.598	0.92 (0.69–1.24)
Intercept		1.01	0.456	2.73 (0.19–38.42)

This summary includes the estimate, *P-*value, and odds ratio (95% confidence interval). * indicates significant *P-*value as alpha = 0.05.

**Table 5:** Summary of the models created to assess the role of geographic location and flea clade, host *B*. *clarridgeiae* infection, and flea coinfection in *Ctenocephalides felis B*. *clarridgeiae* infection when cat sex, weight, and number of fleas are controlled for.

		**Estimate**	***P*-value**	**OR (95% CI)**
**Geographic location and flea clade model**				
Geographic location	Raleigh, NC	Ref	Ref	Ref
	Washington, NC	−0.78	0.36	0.46 (0.09–2.43)
	Orange, VA	−0.52	0.96	0.95 (0.14–6.34)
	Madison, WI	0.21	0.91	1.24 (0.03–60.42)
Flea clade	Clade 4	Ref	Ref	Ref
	Clade 1	−0.62	0.64	0.54 (0.04–7.42)
	Clade 6	1.07	0.33	2.93 (0.33–25.88)
Cat sex	Female	Ref	Ref	Ref
	Male	1.44	0.032*	4.22 (1.13–15.70)
Cat weight		−0.89	0.046*	0.41 (0.17–0.98)
Number of fleas		−0.08	0.598	0.92 (0.69–1.24)
Intercept		1.01	0.456	2.73 (0.19–38.42)
**Host** ***Bartonella*** ***clarridgeiae* model**				
Cat infection	Negative	Ref	Ref	Ref
	*Bartonella clarridgeiae*	0.17	0.82	1.19 (0.27–5.15)
Cat sex	Female	Ref	Ref	Ref
	Male	1.41	0.04*	4.11 (1.09–15.54)
Cat weight		−0.86	0.06	0.42 (0.17–1.03)
Number of fleas		−0.08	0.60	0.92 (0.69–1.24)
Intercept		0.90	0.53	2.46 (0.15–39.50)
**Flea coinfection model**				
Flea *Rickettsia*	Negative	Ref	Ref	Ref
	*Rickettsia asembonensis*	−0.57	0.47	0.57 (0.12–2.65)
	*Rickettsia felis*	−17.61	0.99	Null
Flea *Wolbachia*	Negative	Ref	Ref	Ref
	*Wolbachia* spp.	−0.49	0.47	0.61 (0.16–2.34)
Cat sex	Female	Ref	Ref	Ref
	Male	1.62	0.02*	5.04 (1.30–19.50)
Cat weight		−0.86	0.06	0.42 (0.17–1.05)
Number of fleas		−0.10	0.50	0.90 (0.66–1.22)
Intercept		1.81	0.31	6.14 (0.18–204.07)

This summary includes the estimate, *P-*value, and odds ratio (95% confidence interval). * indicates significant *P-*value as alpha = 0.05. Null indicates no fleas were in that category and therefore an OR could not be calculated.

## 4. Discussion

This study found that *Rickettsia asembonensis* (62%) and *Bartonella clarridgeiae* (19%) were the most common pathogenic bacteria detected in fleas. *R*. *asembonensis* was detected exclusively in Clade 4 *C*. *felis* regardless of geographic location yet was only detected in one single cat. *Bartonella clarridgeiae* infection was detected in a similar proportion of *C*. *felis* (21%) and cat hosts (17%), but *C*. *felis* infection was only independently associated with cat sex (higher in male cats) and weight (higher in lighter cats), and not with cat infection status, geographic location, or flea clade.

*Bartonella clarridgeiae* was the most common *Bartonella* spp. infecting fleas and cats in this study. Interestingly, only cat host demographic factors (cat sex and body weight), and not flea factors such as flea clade or co-infection, nor cat *Bartonella* infection status were significantly independently associated with flea *B*. *clarridgeiae* infection. Unlike *B*. *clarridgeiae*, *B*. *henselae* and *B*. *koehlerae* were only detected in Clade 4 *C*. *felis*. Based upon previous studies detecting *B*. *henselae* in Clades 1, 3, and 6, and *B*. *koehlerae* in Clades 1 and 4, it is likely that these *Bartonella* spp., are not strictly clade specific (Šlapeta and Šlapeta, 2016; Manvell et al., 2022). Our failure to detect *B*. *henselae* in the Clade 1 and 6 fleas collected for this study may be due to relative rarity of infection in these clades combined with small sample size of Clade 1 (*n* = 17) and Clade 6 (*n* = 7) fleas or restriction of sampling sites to the eastern and midwestern United States. Further sampling of wild-caught fleas, as well as laboratory studies assessing the efficiency of *Bartonella* spp. acquisition by *C*. *felis* from diverse genetic backgrounds and coinfection status are warranted.

Despite our finding that cat sex and body weight were independently associated with flea *B*. *clarridgeiae* infection, there was poor agreement between individual cat and flea *Bartonella* spp., infection status. Only three of 10 *Bartonella* infected cats hosted fleas infected with the same *Bartonella* spp. In agreement with previous publications, we concluded that fleas collected from a specific cat do not provide insight into the *Bartonella* infection status of that cat (Chomel et al., 1996; La Scola et al., 2002; Gutiérrez et al., 2015; Mifsud et al., 2020). Furthermore, a higher percentage of *Bartonella* spp. uninfected (qPCR negative) cats (30%, 43/145) had fleas collected than *Bartonella* spp. infected cats (23%, 10/44). This finding reinforces that it is not necessary for cats to present with flea infestation to consider the possibility of *Bartonella* spp. infection. The inability for study personnel to collect fleas from *Bartonella* spp. infected cats may be indicative of highly efficient grooming by the cat host or the long duration of *Bartonella* infection in the cat, an important characteristic of their role as a reservoir host (Kordick and Breitschwerdt, 1988; Taber et al., 2022).

Flea *Rickettsia* spp. infection was significantly associated with flea phylogenetic clade. *Rickettsia felis* DNA was amplified only in Clades 1 (32%, 6/19) and 6 (20%, 1/5) while *R*. *asembonensis* was amplified from almost all *C*. *felis* from Clade 4 (96%, 52/54) but no *C*. *felis* from Clades 1 or 6. This clade specificity was previously reported in a larger sampling of *C*. *felis* from California, Louisiana, and North Carolina which included a portion of the fleas reported in this manuscript (Manvell et al., 2022). The detection of *Rickettsia* spp. in the tissue of 9% (5/56) of cats from Raleigh, NC was surprising considering that we did not detect *Rickettsia* spp. in cats from any other location (*n* = 133 cats). Some studies have failed to detect *Rickettsia* spp. in cats when performing PCR on blood (Hawley et al., 2007; Barrs et al., 2010); however, other studies report comparable infection rates to those found in Raleigh, NC (Mullins et al., 2018; Phoosangwalthong et al., 2018). The diversity of these *Rickettsia* spp. is of interest as we detected DNA sequences of two *C*. *felis* associated *Rickettsia* spp. (*R*. *asembonensis* and *R*. *felis*) and an unnamed *Rickettsia* spp. genetically most similar to *Rickettsia* spp. reported in ticks (Lee et al., 2014). Investigation of the relative clinical importance of these *Rickettsia* spp. for cats, humans, and other animals is warranted.

Our data supports the use of fleas as sentinels for population level cat *Bartonella* spp. presence and relative abundance. Within individual geographic locations, we reported complete agreement between fleas and cats for the presence of *B*. *clarridgeiae*, *B*. *henselae*, and *B*. *koehlerae*. *Bartonella clarridgeiae* was the most common *Bartonella* spp. in both cats and fleas from all locations except Washington, NC. Washington, NC displayed the highest proportion of *B*. *henselae* infection in both cats and fleas and was the only location where *B*. *henselae* was more common than *B*. *clarridgeiae* in fleas. Washington, NC was also the only location with documented *B*. *koehlerae*, which we found in both cats and fleas. Our data supports that flea sampling can provide insight into the *Bartonella* species diversity and relative abundance in a specific geographic area. This information is critical for regional diagnostic considerations and for the prevention of *Bartonella* spp. transmission to aberrant hosts (human, dog, etc) in which clinical manifestations are typically more severe and cryptic, with lower bacteremia hindering diagnosis.

The detection of *C*. *simplex*, *N*. *fasciatus*, and *O*. *howardi* infesting cats indicates the need for further investigation of the regional diversity of fleas infesting cats, the ability of these fleas to transmit pathogens, and the efficacy of flea control products for the control of these flea species. Each of these non-*C*. *felis* fleas was collected from a different cat, confirming that infestation of non-*C*. *felis* fleas on cats has occurred multiple times in this community. The capacity of these fleas to serve as a vector for disease transmission is essentially unknown. In this small sample size one *C*. *simplex* was infected with *B*. *alsatica* (20%, 1/5). This finding is of potential relevance to human medicine, as *B*. *alsatica*, a rabbit reservoir adapted species, has been reported in cases of endocarditis and host versus graft rejection (Raoult et al., 2006; Jeanclaude et al., 2009; Puges et al., 2019). *Bartonella*, hemotropic *Mycoplasma*, and *Rickettsia* spp. DNA was not amplified from *N*. *fasciatus* (*n* = 1) or *O*. *howardi* (*n* = 1). This may be due to a lack of vector competence, the presence of these flea species on atypical hosts that are not as efficient in maintaining and transmitting associated pathogens, or the small number of fleas tested in this study.

The most prevalent *Wolbachia* strain (wCfeT) did not display *C*. *felis* clade specificity, with detection in all clades sampled (1, 4, and 6), while other *Wolbachia* strains (wCfeF, wCfeJ, and an uncharacterized strain) were only detected in Clade 6. We propose that this finding may be related to increased diversification and evolution of *Wolbachia* within certain *C*. *felis* clades. *Wolbachia* species are a known endosymbiont of most insect species with certain strains causing profound effects in the species they infect (Werren et al., 2008). Genomic comparison of two *C*. *felis* associated *Wolbachia* (wCfeT and wCfeJ) strains indicated differential biotin synthesis, as well as cytoplasmic incompatibility-like genes with unknown implications for fleas or flea-borne pathogens (Driscoll et al., 2020). Flatau et al. (2018) reported that increased *Wolbachia* loads lowered reproductive success in *Synosternus cleopatrae*, a flea species infecting gerbils. This effect was observed only in laboratory fleas and not wild-caught fleas indicating the specificity of these associations which may be due to flea genetic diversity or variations in the flea associated microbiome. Further exploration of *C*. *felis*-*Wolbachia* relationships is necessary to define the evolutionary advantages or disadvantages conveyed by specific *Wolbachia* strains, as well as the ability of diverse *C*. *felis* clades to acquire and maintain various *Wolbachia* strains and the implications for flea-borne pathogen transmission.

We did not amplify hemotropic *Mycoplasma* spp. from the fleas in this study despite flea collection from hemotropic *Mycoplasma* infected cats, a finding also reported by other studies (Persichetti et al., 2016; Abdullah et al., 2019). It is unknown if this is due to the geographic location sampled, lack of flea pooling, or other methodological differences.

One major limitation of this study was that fleas were not collected from every cat that a tissue sample was obtained from. While the flea collection protocol was standardized, since the aim of the study was not to determine the prevalence of flea infestation in this cat population it is likely that fleas from cats with lower flea burdens or cats with fleas that were difficult to catch were underrepresented in this sample. The length of time cats were housed in traps prior to anesthesia may have also impacted whether fleas were able to be collected, since fleas may not have been found on cats with longer waiting times or more efficient groomers. Because of this uncertainty, we did not attempt to investigate flea presence or flea burden on individual cats as explanatory factors for flea-borne pathogens. Additionally, our small sample of non-*C*. *felis* fleas and *C*. *felis* from Clades 1 and 6 prevented a thorough investigation of the pathogen occurrence and prevalence of pathogen coinfection in these fleas. Samples were collected at different times from different locations, a potentially confounding factor for geographic location-based conclusions. Furthermore, our small sample of *R*. *felis* and *B*. *henselae* infected fleas prevented the application of a modeling approach for analysis of potential host and flea factors associated with infection. Our means of pathogen detection (qPCR) has imperfect sensitivity for detection of stealth pathogens (such as *Bartonella* spp.), likely resulting in under reporting infection in cat tissues and potentially fleas (Maggi et al., 2020; Lashnits et al., 2021). The collection of tissue and not blood samples may restrict comparison to other publications as tissue displays higher *Bartonella* spp. sensitivity by qPCR (Lashnits et al., 2021). Genus-specific PCR, such as the assays employed herein, are unable to efficiently detect coinfection with more than one species within the genus due to preferential amplification, so we were unable to evaluate coinfection in a single sample (flea or cat), a phenomenon known to occur with flea *Wolbachia* spp. (Driscoll et al., 2020).

In conclusion, our study documented a significant independent association of flea *B*. *clarridgeiae* infection with cat sex and body weight. The cause of this findings is currently unknown and warrants investigation. The lack of agreement between specific cat host and flea pairs was expected on the basis of previous literature; however, geographical correlations of *Bartonella* spp. presence supports the use of fleas as sentinels to detect the relative proportion of *Bartonella* spp. in circulation within specific geographic areas. The detection of fleas not traditionally associated with cats (*C*. *simplex*, *O*. *howardi*, and *N*. *fasciatus*) raises questions regarding the importance of these flea species as a cause of allergy or as vectors for pathogen transmission. The association of *Rickettsia* spp. with specific flea clades should encourage future research regarding flea phylogenetic and coinfection associations with flea-borne pathogen prevalence. Finally, the association of *Bartonella* spp. with *R*. *asembonensis* infection and *C*. *felis* genetic diversity should inspire future investigation of the effect of coinfection and vector diversity in pathogen acquisition and maintenance by this ubiquitous vector.

## Data availability statement

The datasets presented in this study can be found in online repositories. The names of the repository/repositories and accession number (s) can be found at: https://doi.org/10.5061/dryad.0k6djhb43.

## Ethics statement

The animal study was reviewed and approved by North Carolina State University IACUC protocol #19–003 and #21–468, and University of Wisconsin IACUC protocol #V006461.

## Author contributions

CM, EB, and EL conceived and designed experiments, and acquired funding. CM and EL performed data curation, formal analysis, software development, data visualization, and original draft writing. CM, LK, YL, KF, RM, and EL performed sample collection and processing. KF, EB, RM, and EL performed project supervision and administration. All authors reviewed and approved the final manuscript.

## Funding

This research was supported by the State of North Carolina and donations to the North Carolina State University College of Veterinary Medicine Bartonella Vector Borne Diseases Research Fund. A portion of this project was completed while CM was supported by the North Carolina State University’s Comparative Medicine Institute Summer Interdisciplinary Research Initiative and the NC State Molecular Biotechnology Training Program of the National Institutes of Health under award number 1T32GM133366. Portions of this project were also completed while EL research was supported by the Comparative Medicine and Translational Research Program of the National Institutes of Health under award number T32OD011130 and the University of Wisconsin School of Veterinary Medicine Companion Animal Fund.

## Conflict of interest

In conjunction with S. Sontakke and North Carolina State University, EB holds US Patent No. 7,115,384 Media and Methods for Cultivation of Microorganisms, which was issued on October 3rd, 2006, and also co-founder, shareholder, and Chief Scientific Officer for Galaxy Diagnostics, a company that provides advanced diagnostic testing for the detection of *Bartonella* spp. infections.

The handling editor MA declared a past co-authorship with the EB and RM.

The remaining authors declare that the research was conducted in the absence of any commercial or financial relationships that could be construed as a potential conflict of interest.

## Publisher’s note

All claims expressed in this article are solely those of the authors and do not necessarily represent those of their affiliated organizations, or those of the publisher, the editors and the reviewers. Any product that may be evaluated in this article, or claim that may be made by its manufacturer, is not guaranteed or endorsed by the publisher.
