# Supplementary material for: The association of host and vector characteristics with Ctenocephalides felis pathogen and endosymbiont infection
Source: Front Microbiol. 2023 Mar 6;14:1137059. doi: 10.3389/fmicb.2023.1137059 (PMC10025546; doi:10.3389/fmicb.2023.1137059)
Supplement: Supplementary file 1 [file Image_1.pdf]

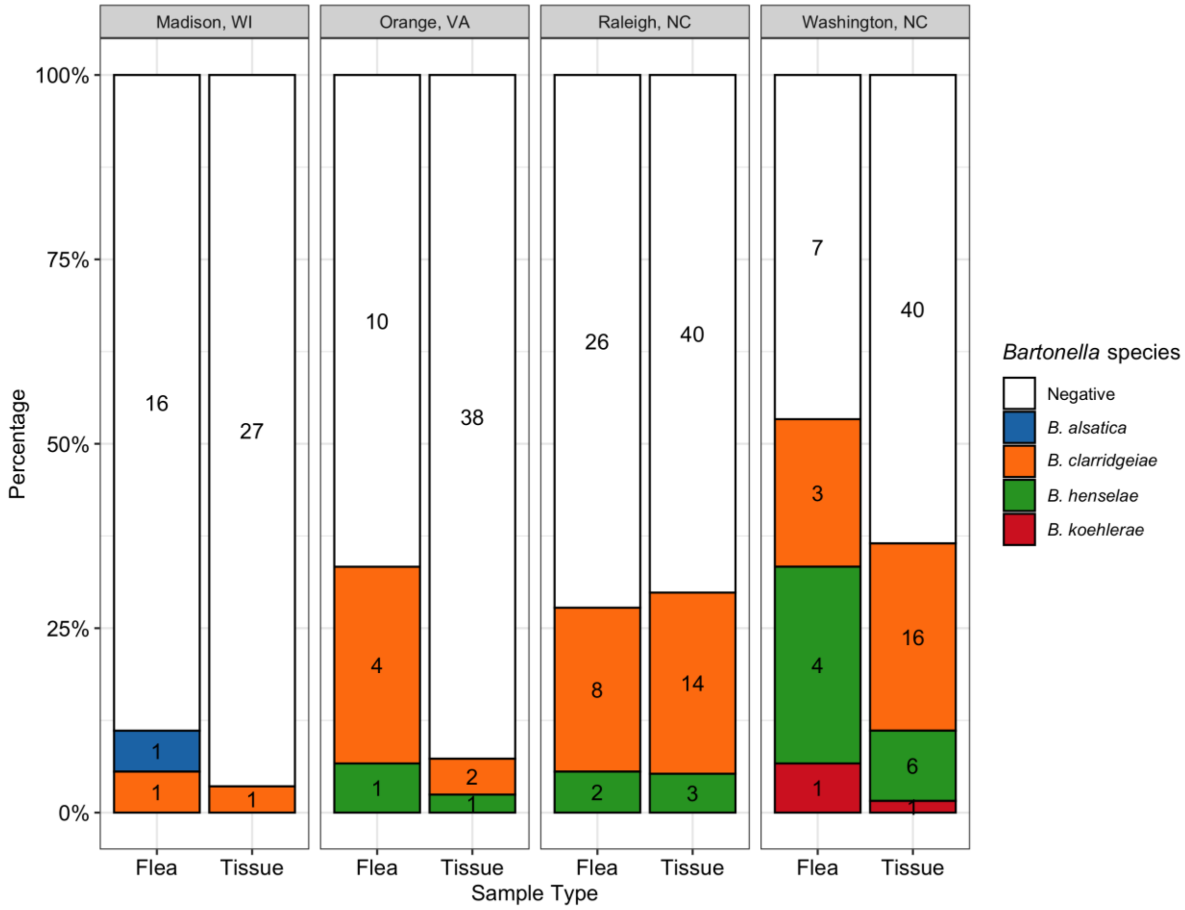

Supplementary Figure S1 percentage of cats and fleas from each location (x-axis) infected with *Bartonella* spp. (color). The total number of cats or fleas in each category is indicated.
